# An adaptive predefined time sliding mode control for uncertain nonlinear cyber-physical servo system under cyber attacks

**DOI:** 10.1038/s41598-024-57775-8

**Published:** 2024-03-28

**Authors:** Saleem Riaz, Bingqiang Li, Rong Qi, Chenda Zhang

**Affiliations:** Present Address: School of Automation, Northwestern Polytechnical University, Xi’an, 710072 China

**Keywords:** Cyber-physical systems (CPS), Seeker servo control, Nonlinear control, Uncertain control systems, Cyber attacks, Robust control, Engineering, Electrical and electronic engineering

## Abstract

Malicious attacks are often inevitable in cyber-physical systems (CPS). Accuracy in Cyber physical system for position tracking of servos is the major concern now a days. In high precision industrial automation, it is very hard to achieve accuracy in tracking especially under malicious cyber-attacks, control saturations, parametric perturbations and external disturbances. In this paper, we have designed a novel predefined time (PDT) convergence sliding mode adaptive controller (PTCSMAC) for such kind of cyber physical control system. Main key feature of our control is to cope these challenges that are posed by CPS systems such as parameter perturbation, control saturation, and cyber-attacks and the whole system then upgrade to a third-order system to facilitate adaptive control law. Then, we present an adaptive controller based on the novel PDT convergent sliding mode surface (SMS) combined with a modified weight updated Extreme Learning Machine (ELM) which is used to approximate the uncertain part of the system. Another significant advantage of our proposed control approach is that it does not require detailed model information, guaranteeing robust performance even when the system model is uncertain. Additionally, our proposed PTCSMAC controller is nonsingular regardless of initial conditions, and is capable of eradicating the possibility of singularity problems, which are frequently a concern in numerous CPS control systems. Finally, we have verified our designed PTCSMAC control law through rigorous simulations on CPS seeker servo positioning system and compared the robustness and performance of different existing techniques.

## Introduction

Many industries has reported significant cyberattacks recently such as BlackEnergy event in Ukraine^1,2^, the WannaCry ransomware have brought attention for the researchers to develop resilient control technology. The similar cases have been occurred in other industries such as health care^3,4^, economics and energy sectors^5^. Healthcare, transportation, and industrial automation are just a few of the sectors that have seen revolutionary advancements since due to the rapid expansion of Cyber-Physical Systems (CPS)^6,7^. Considering their critical significance in high-risk industries like aerospace and complex manufacturing, Seeker Coordinator Servo Control systems have evolved as a particularly important subgroup among the variety of applications^8–10^. To address the rising demands for accuracy and responsiveness, traditional control paradigms, which frequently rely on linear models, have increasingly made way for more complex strategies like second-order nonlinear control^11–13^. The switch to more intricate control structures is not without its difficulties, though. Second-order nonlinear control offers advantages in modeling accuracy, but these systems are complicated and prone to a variety of weaknesses such as malicious attacks in CPS systems^14–18^. It is essential to build control techniques that are not only accurate but also robust since system uncertainties, such as sensor noise and actuator failures, can dramatically reduce performance^19,20^. Additionally, as industrial systems become more networked, the attack surface increases and they become more appealing targets for cyberattacks^21^. A cyber-attack might have disastrous effects since Seeker Coordinator Servo Control systems are frequently used in situations where failure is not an option, such missile guiding systems. This calls for the creation of control techniques that are both reliable and secure. There is a pressing need for control techniques that can survive both uncertainty and malicious threats, as described in^22,23^.

Although there is broad range of study on friction modeling and dynamic compensating control technology. It did not until the 1980s that research in this field started to gain pace because of constraints in control theory and the condition of basic scientific development at the time. Currently, servo system interference is evaluated and combated using a mix of identification and compensation. Strategies for reducing or adjusting for friction torque are frequently used to test and locate interference sources in the search of a more accurate model for the seeker coordinator system. In order to successfully reduce the effect of friction torque on identification outcomes, a unique frequency-domain identification approach that makes use of a square wave signal excitation system was presented in^24^. To reduce the negative impacts of friction torque,^25^ identifies the system model after friction torque adjustment. The introduction of an ESO based Kalman filter to approximate friction is another novelty in^26,27^, and it considerably improves the performance of the servo system. There are LuGre as well as Stribeck model are the two most often used friction disturbance models recently^28–31^. The pre-slip stage is not well described or transitioned by the Stribeck friction model, even though it accurately describes friction torque during the sliding stage. The dynamic and static characteristics of friction are thoroughly explained by the LuGre model, in contrast, using the theory of bristle deformation, providing a more accurate depiction of ground sliding and pre-slip phases. However, due to the complex nature of its modeling and identification methods, its practical implementation in engineering is deferred. Second-order nonlinear control is used to simulate real-world dynamics more accurately, but it also introduces several complexity and drawbacks especially when it comes under malicious attacks. Systems uncertainties such sensor noise and actuator failures, on the other hand, continue to be a continual problem^32–34^. Besides these challenges, such kind of systems have become more popular and connected, making them valuable targets for cyberattacks such advanced persistent threats and malicious attacks^35,36^. Given these difficulties and the crucial nature of the applications at hand, this study seeks to make numerous significant contributions to the body of knowledge already in existence. To the best of our knowledge some researchers unable to get high precision and fails to implement under cyber-attacks^37–39^, especially when the case is trajectory tracking control of a practical servo coordinator control system. Also, these above-mentioned techniques have been designed by using fixed and finite time control approach. In fact, CPS systems such as industrial control, power networks, flight control , online life prediction of lithium-ion batteries and data driven approaches are prone to vitriolic attacks^40,41^. What is the main key issue is that design a resilient control and give the stability criteria in predefined. So that control law will be able to adaptively cope these malicious attacks as well as multiple uncertainties.

Recent robust control strategies in CPS, especially for PMSM, are studied and demonstrated to profoundly rely on asymptotic stabilization, which may result in infinite convergence times. To accelerate convergence, finite time control algorithms, such as comprehensive super twisting observer and backstepping method-based control laws, are introduced. Though, these algorithms often overlook parameter uncertainty and external interference, affecting their robustness^42^. In order to cope this robustness problems, a sliding mode control combined with finite time control has shown promising trajectory tracking, with developments like non-singular terminal sliding law and observer-based algorithms guaranteeing finite time convergence of the motor's position^43^. Apart from these advancements, the convergence time of these systems can be unpredictable due to dependencies on initial state values and control parameters, leading to the development of fixed-time convergence control algorithms, which set the maximum convergence time based on control parameters and have been applied to various systems including wheeled robots and dynamic positioning ships^44–48^. It can be concluded from above analysis that existing control strategies for Cyber-Physical Systems (CPS) are primarily limited by their robustness on model information, leading to challenges in universality and adaptability with CPS updates. Such kind of conditions underlines that there is lack of adaptive control strategies to be proposed. Moreover, such methods are only ensure asymptotic convergence without PDT guarantees, limiting the practical application of finite time/fixed time convergence schemes. Traditional and existing SMC laws commonly used for CPS safety, also falls short as it does not allow for presetting convergence times, leading to unpredictability in system response and a need for more dynamic and robust control mechanisms for CPS security. There is a new tactic called predefined time stability which is the main contribution of this paper. It is studied that neither control parameters nor initial conditions have impact on the upper bound of convergence time, though this study still requires further research due to limited existing knowledge. Therefore, we have designed a novel predefined time (PDT) convergence sliding mode adaptive controller (PTCSMAC) and multiple case studies considered for PMSM seeker coordinator control CPS system under malicious attacks as well as multiple uncertainties including input saturation and parametric perturbations.

Our main scientific contributions are summarized as follows:

- A novel predefined time (PDT) convergent sliding surface is designed in this paper. Then derived a robust adaptive control mechanism based on this PDT sliding surface and given Lyapunov stability theory. This novel adaptive PTCSMAC control law uses modified weight updated extreme learning machine criterion intended to effectively reduce the impact of uncertainties and cyber-attacks.
- Apart from the fixed and finite time sliding mode control laws, we have designed a novel predefined time (PDT) sliding surface for PMSM seeker servo position control under different cyber-attacks. The nonsingular design of our proposed controller eliminates risks of singularity issues in control systems and the results are compared with other existing control techniques as depicted in^37,38^.
- Significantly, the initial states of the system are independent of the established control law, allowing for their arbitrary selection within a predefined time region. This means that initial conditions are not influenced or restricted by the control mechanism that has been developed, offering flexibility in setting these initial states as long as they fall within the predetermined boundaries of the operational parameters of controlled system.
- A comprehensive simulation analysis is given for the reliability and robustness of proposed control law. Both security and performance are checked under challenging circumstances such as uncertainties and cyber-attacks and multiple uncertainties. Results determined that our proposed robust control can mitigate the impact of these uncertainties and malicious attacks as compared to existing control laws as given in^37–39^.

The rest of this paper is structured as follows: "System Modeling and Problem Formulation", first we have discussed the main key challenges, topic relevance, background of the proposed study, and then formulates the main problem with cyber-attack categories which offers a whole CPS mathematical model of the seeker coordinator servo system. The modified weight updated ELM based control technique, which combines Lyapunov-based control law with a sophisticated disturbance detection system (system extended state), is further described in "Predefined time convergence stability criteria and sliding surface design". The simulation results under different cyber-attack cases are presented in "Design of PDT sliding mode controller for CPS servo system", which provides empirical support for the Cyber-Physical Control System for Seeker Coordinator Servo Nonlinear Positioning System. The work is concluded in "Simulation results and analysis", which summarizes the major conclusions and presents potential directions for further investigation.

## System modeling and problem formulation

### Seeker coordinator mathematical model

The next development in the merging of the digital and physical domains is represented by Cyber-Physical Systems (CPS), which allows for advanced automation and control in a range of applications. Advanced guidance systems, where a ground command center has exact control over a remote seeker, are a prime illustration of CPS in action. This integration, which combines physical processes with computing aspects (cyber), embodies the essence of CPS. A seeker is an example of the cyber component of CPS and is controlled by wireless commands. It gets commands from the ground command center, which creates a complex information physics system by carefully processing data and sending out control signals. A strong network that guarantees real-time communication and control even over great distances connects the seeker and the command center.

**Figure 1:**
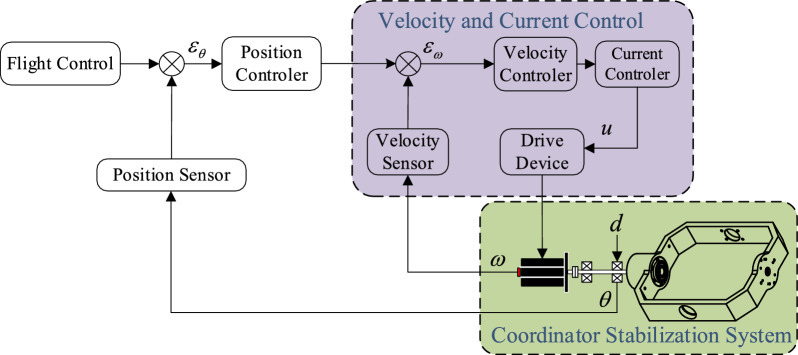
Seeker coordinator stabilization system’s Model diagram.

The Permanent Magnet Synchronous Motor, or PMSM, is the engine that powers the seeker's motion and accuracy. The PMSM is well known for its exceptional torque-weight ratio, high efficiency, and superior controllability. Because of these qualities, it is the perfect choice for applications requiring a high degree of accuracy and dependability, including seeker control. Because of the PMSM's connection with the CPS framework, the seeker's trajectory may be carefully adjusted to ensure precise positioning and maneuvering in accordance with the guiding head's instructions from the command center. As the CPS's central decision-making unit, the guidance head interprets sensor input and converts it into commands that may be carried out. The guidance head may apply intricate algorithms to precisely regulate the seeker's orientation, speed, and position by utilizing the PMSM's capabilities. This mutually beneficial interaction demonstrates how CPS may improve the operational efficiency of distant systems, opening the door for developments in precise aiming and autonomous navigation. The combination of PMSM and CPS spurs innovation in control systems and signifies a time when digital intelligence will easily coordinate physical objects in a world that is becoming more interconnected. PMSM has been extensively applied in aircraft, industrial and other high-precision systems, it has several great outstanding characteristics including superior effectiveness, low noise, enhanced power density, small-size and many more. PMSM is selected as the driving mechanism of seeker coordinator stabilization system. By driving the PMSM through the controller, the high-precision control is accomplished in seeker coordinator control as shown in Fig. 1.

The pitch axis mathematical model description of PMSM seeker coordinator servo is elaborated with combination of electrical and combined mechanical equations as follows:

1 $$\left\{ \begin{gathered} U = R \cdot I + L \cdot \frac{dI}{{dt}} + K_{e} \cdot \omega \hfill \\ T_{e} = 1.5p\psi_{f} I\,\,\,\,\,\,\,\,\,\, \hfill \\ J\frac{d\omega }{{dt}} = T_{e} - B_{m} \omega - \frac{{T_{L} }}{{n_{g} }} - T_{d} \hfill \\ \end{gathered} \right.$$

where, $(K_{T} = 1.5p\psi_{f} )$ , *U* is the stator voltage. *I* represent armature current while $n_{g}$ denotes the transmission gear ratio. *R* is armature resistance. $\theta$ and $\omega$ represent the rotor mechanical angle and angular velocity output. *T*_*e*_ is the motor’s electromagnetic torque. *L* is armature inductance. *J* is the sum of the equivalent moment of inertia. *B*_*m*_ is the viscous damping coefficient on the motor. *K*_*T*_ is the electric torque coefficient. *K*_*e*_ is the back EMF coefficient. $T_{L}$ is the external disturbance load torque (N.m) including friction force and load disturbance torque. In low-speed operation, when the servo system's tracking performance is greatly reduced and prone to creeping behavior, this impact is particularly noticeable. As a result, external disturbances are divided into two groups: other disturbances such as ripple torque and friction torque disturbances.

2 $$T_{d} = T_{f} + T_{ripple}$$

where *T *_*f*_ depicts the frictional torque while $T_{ripple}$ denotes the ripple torque disturbance.

Pulsating torque and friction torque signify the main disturbance in the seeker coordinator stabilization system. The following equation represents the pulsating disturbance torque as a result of the cogging effect in the PMSM:

3 $$T_{ripple} = \sum\limits_{i = 1}^{N} {A_{Ri} \sin (\omega_{i} t + \Phi_{i} )}$$

where $A_{R}$ is the amplitude and $\Phi_{i}$ is the phase angle.

Though the friction torque model is commonly complicated, it is typically associated to the frame angle and velocity. Using Stribeck model in mathematical form has been elaborated as follows:

4 $$T_{f} = k_{v} \omega + f_{c} {\text{sgn}} \left( \theta \right) + \left( {f_{s} - f_{c} } \right){\text{e}}^{{ - \left( {\frac{\omega }{{\dot{\theta }_{s} }}} \right)^{2} }}$$

In above formula, *k*_*v*_ is a viscous friction coefficient, *f*_*c*_ means the kinetic friction, *f*_*s*_ symbolizes the static friction, $\dot{\theta }_{s}$ designates the critical velocity.

The analysis of system model as explored in reference^49^, focused primarily on the fixed-time stability of closed-loop Cyber-Physical Systems (CPSs) in the absence of malicious cyber activities and external disturbances. However, it's crucial to recognize that, in real-world scenarios like Internet of Things (IoT), aviation control systems, electrical networks, and water distribution systems, the presence of cyber threats and external interferences is an unavoidable reality. The challenge is significantly amplified when these hostile cyber incursions and external disruptions co-occur, making the achievement of fixed-time stability in higher-order nonlinear CPSs notably more complex. Consequently, the approach outlined in^49^ becomes ineffective in ensuring fixed-time stability under these conditions. Addressing this issue and ensuring fixed-time stability in the face of these complicating factors represents a substantial and non-trivial challenge.

In practical engineering applications, the current input $i_{q}$ value is bounded, so controlling the input $u(t)$ the following condition must hold:

5 $$|u(t)| \le u_{{\text{M}}}$$

On the other hand, in cyber physical systems, the control of the seeker is transmitted to PMSM through the network, and during the network transmission process, the control input is easily attacked by malicious networks. All malicious network attacks can be divided into multiplicative and additive attacks. Define the system state variables in (1) as $$x = \left[ {\begin{array}{*{20}c} {x_{1} } & {x_{2} } \\ \end{array} } \right]^{T} = \left[ {\begin{array}{*{20}c} \theta & \omega \\ \end{array} } \right]^{T}$$, $u(t) = i_{q} (t)$ then the PMSM model affected by network attacks is:

6 $$\left\{ \begin{gathered} \dot{x}_{1} (t) = x_{2} (t) \hfill \\ \dot{x}_{2} (t) = = - \frac{{B_{m} }}{J}x_{2} (t) + \left( {\frac{{K_{T} }}{J}} \right)\left( {\alpha \left( {t_{m} } \right)u(t) + \beta \left( {t_{a} } \right)} \right) - d(t) \hfill \\ y(t) = x_{1} (t) \hfill \\ \end{gathered} \right.$$

Among them, $d(t) = \frac{{T_{L} }}{{n_{g} J}} + \frac{{T_{d} }}{J}$ is the overall disturbance torque, $\beta (t_{a} )$ represents an additive attacking signal, and its value is given as:$\beta (t_{a} ) = 0.003 + 0.001{\text{e}}^{0.1t}$, here $t_{a}$ denotes the instant when additive attack signal happens. While $\alpha \left( {t_{m} } \right) \in (0,\infty )$ denotes Multiplicative actuation attacks, whose value is taken as $\alpha \left( {t_{m} } \right) = \cos^{2} \left( {x_{2} } \right)$ and $t_{m}$ is the time instant when the multiplicative actuation attacks will happen.

**Figure 2:**
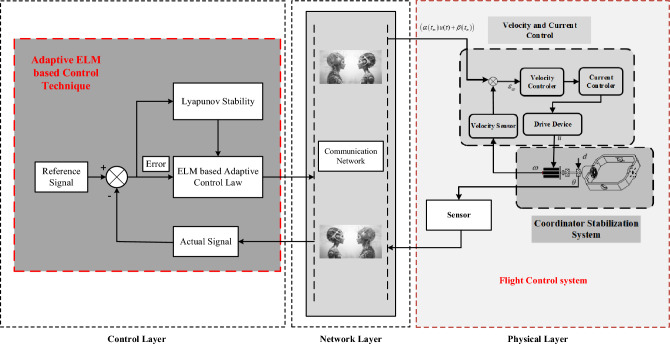
ELM based adaptive predefined time seeker coordinator CPS control.

The overall control structure with the proposed mathematical model for seeker servo CPS adaptive position control at low speeds is given in the following Fig. 2.

#### Assumption 1

As described in^50^, suggests that the uncharacterized time-variable functions $\alpha \left( {t,t_{m} } \right)$ and $\beta (t,t_{a} )$ are bounded. This implies the existence of positive constants $\alpha_{\min }$ and $\beta_{\max }$,which set the bounds for these functions. Specifically, $\alpha \left( {t,t_{m} } \right) < \alpha_{\min }$ and the absolute value of $\beta (t,t_{a} )$ is always less than or equal to $\beta_{\max }$($\beta (t,t_{a} ) \le \beta_{\max }$). This assumption ensures that these time-varying functions remain within specified limits over time.

As described in above assumption 1, both the multiplicative actuation attack, denoted as $\alpha \left( {t,t_{m} } \right)$ and the uncontrollable additive attack signal $\beta (t,t_{a} )$ are supposed to be unknown entities. This uncertainty renders traditional fixed-time stability criteria inapplicable. Specifically, the multiplicative actuation attack $\alpha \left( {t,t_{m} } \right)$ symbolizes a stealth attack, characterized by its covert nature and ability to manipulate system operations without detection. On the other hand, the additive attack signal $\beta (t,t_{a} )$ signifies a deception attack, which is designed to mislead or corrupt data of the system and processing, leading to inaccurate operations or interpretations.

If there is a constraint $u(t)$ for the input $|u(t)| \le u_{{\text{M}}}$, and the unconstrained input voltage is $v(t)$, then the deviation between the unconstrained input $v(t)$ and the constrained input $u(t)$ is $\theta (t)$, then there is:

7 $$v = u + \theta$$

where $\theta (t)$ can be represented as follows:

8 $$\theta (t) = \left\{ \begin{gathered} 0\;\;\;\;\;\;\;\;\;\;\;\;\;\;\;\;\;\;\;\;\;\;|v| < u_{{\text{M}}} \hfill \\ v - {\text{sign}} (v)u_{{\text{M}}} \;\;\;\;\;|v| \ge u_{{\text{M}}} \hfill \\ \end{gathered} \right.$$

Obviously, the deviation $\theta (t)$ is bounded.

By considering the existence of parameter perturbations in PMSM seeker servos we have assumed that the parameters are taken in the following way such as: $$M = - \frac{{B_{m} }}{J}$$, $$N = \left( {\frac{{K_{T} }}{J}} \right)$$, $$d(t) = \frac{{T_{L} }}{{n_{g} J}} + \frac{{T_{d} }}{J}$$ and then we have $$M = M_{0} + \Delta M,N = N_{0} + \Delta N$$. In practical engineering applications, the nominal parameters can be calculated by measuring the physical properties of the motor, so the actual variables $$M,N$$ are decomposed into the sum of its nominal variable $$M_{0} ,N_{0}$$ and these variables have perturbation of $$\Delta M,\Delta N$$ such that $$M = M_{0} + \Delta M,$$$$N = N_{0} + \Delta N$$. Substitute $$M = M_{0} + \Delta M,N = N_{0} + \Delta N$$ and $$u = v - \theta$$ into Eq. (6), we can get the following overall system.

9 $$\left\{ \begin{gathered} \dot{x}_{1} (t) = x_{2} (t) \hfill \\ \dot{x}_{2} (t) = Mx_{2} (t) + N\left( {\alpha \left( {t_{m} } \right)u(t) + \beta \left( {t_{a} } \right)} \right) - d(t) \hfill \\ y(t) = x_{1} (t) \hfill \\ \end{gathered} \right.$$

10 $$\Rightarrow \left\{ \begin{gathered} \dot{x}_{1} (t) = x_{2} (t) \hfill \\ \dot{x}_{2} (t) = M_{0} x_{2} (t) + \Delta Mx_{2} (t) + N_{0} u + \Delta Nu + N\left( {(\alpha - 1)u + \beta } \right) - d(t) \hfill \\ y(t) = x_{1} (t) \hfill \\ \end{gathered} \right.$$

11 $$\left\{ \begin{gathered} \dot{x}_{1} = x_{2} \hfill \\ \dot{x}_{2} = M_{0} x_{2} + N_{0} v + G \hfill \\ y = x_{1} \hfill \\ \end{gathered} \right.$$

Among them, $G = \Delta Mx_{2} - N_{0} \theta + \Delta N(v - \theta ) + d(t) + N\left( {(\alpha \left( {t_{m} } \right) - 1)u + \beta \left( {t_{a} } \right)} \right)$ is the composite interference of system parameter perturbation and external interference. If the expected position trajectory of the CPS seeker servo system is $y_{{\text{d}}}$, then the tracking error and its derivative between the expected trajectory and the output of the CPS system are as follows:

12 $$\begin{gathered} e_{1} (t) = y(t) - y_{d} (t) = x_{1} (t) - y_{d} (t) \hfill \\ e_{2} (t) = \dot{e}_{1} (t) = \dot{y}(t) - \dot{y}_{d} (t) = x_{2} (t) - \dot{y}_{d} (t) \hfill \\ \end{gathered}$$

The TTE model of the CPS seeker coordinator servo system is depicted as follows:

13 $$\left\{ \begin{gathered} \dot{e}_{1} (t) = e_{2} (t) \hfill \\ \dot{e}_{2} (t) = M_{0} e_{2} + N_{0} v + G + M_{0} \dot{y}_{d} - \ddot{y}_{d} \hfill \\ \end{gathered} \right.$$

The main goal is to achieve a satisfactory design of a control input $u(t)$ for our servo system (11)with parameter perturbations, external disturbances, and control saturation, so that the system output $y(t)$ can accurately track the expected trajectory $T_{s}$ within the predefined time $y_{{\text{d}}} (t)$, i.e. $\mathop {\lim }\limits_{{t \to T_{s} }} e_{1} (t) = 0$.

To facilitate system stability analysis, the following theorem is given first.

## Predefined time convergence stability criteria and sliding surface design

### Theorem 1

*Let the nonlinear system* $\dot{x}(t) = f(x),f(0) = 0,x(0) = x_{0}$, *for any PDT* $T_{s} > 0$, *when the positive definite Lyapunov candidate* $V(t)$ *should hold the following condition*:

14 $$\dot{V}(t) \le - \frac{\pi }{{2pT_{s} \sqrt {ab} }}(aV^{1 - p} (t) + bV^{1 + p} (t))$$

In the above expression the respective gains settings are as: $0 < p < 1,a > 0,b > 0$, the nonlinear system $\dot{x}(t) = f(x)$ is a global PDT system, and the upper bound of convergence time is not affected by the initial value of the system and system parameters. It is worth noting that the convergence time meets the following criteria.

15 $$t_{s} = \frac{{2T_{s} }}{\pi }\arctan \left( {\sqrt{\frac{b}{a}} V^{p} (0)} \right) < T_{s}$$

### Proof

According to Eq. (14), the inequality is described as follow:

16 $$\frac{{{\text{d}}V}}{{{\text{d}}t}} \le - \frac{\pi }{{2pT_{s2} \sqrt {ab} }}aV^{1 - p} (1 + \frac{b}{a}V^{2p} )$$

Because of $V \ge 0$, after transforming Eq. (16) and differentiating, we can obtain the following results.

17 $$\frac{1}{{1 + (\sqrt{\frac{b}{a}} V^{p} )^{2} }}{\text{d(}}\sqrt{\frac{b}{a}} V^{p} ) \le - \frac{\pi }{{2T_{s2} }}{\text{d}}t$$

Assuming that the state variable $\dot{x}(t) = f(x)$ of the nonlinear system $x(t)$ satisfies $t \ge t_{s}$ after the time $x(t) \equiv 0$, then there are $x(t_{s} ) = 0$ and $V(t_{s} ) = 0$. Integrating both sides of Eq. (17) yields

18 $$\begin{gathered} \int_{V(0)}^{{V(t_{s} )}} {\frac{{{\text{d(}}\sqrt{\frac{b}{a}} V^{p} )}}{{1 + (\sqrt{\frac{b}{a}} V^{p} )^{2} }}} \le - \int_{0}^{{t_{s} }} {\frac{\pi }{{2T_{s2} }}{\text{d}}t} \hfill \\ \Rightarrow \arctan \left( {\sqrt{\frac{b}{a}} V^{p} (0)} \right) \ge \frac{\pi }{{2T_{s2} }}t_{s} \hfill \\ \Rightarrow t_{s} = \frac{{2T_{s} }}{\pi }\arctan \left( {\sqrt{\frac{b}{a}} V^{p} (0)} \right) < T_{s} \hfill \\ \end{gathered}$$

An upper bound of the CT in the Lyapunov stability criterion of PDT convergent given in Theorem 1 is regardless of the initial value and control parameters of the nonlinear system, so that the CT of the system can be set arbitrarily, effectively improving the controllability of the system's convergence time.

The integral control term in the PID controller plays a major role in eliminating steady-state errors (SSE) in the system. To minimize SSE in seeker coordinator servo position tracking control and expand the position tracking accuracy of the seeker coordinator servo system, construct following higher-order sliding mode :

19 $$\begin{aligned} \sigma = & \ddot{e}_{0} + \frac{\pi }{{p_{1} T_{s1} \sqrt {a_{1} b_{1} } }}(a_{1} (\frac{1}{2})^{{1 - p_{1} }} \dot{e}_{0}^{{1 - 2p_{1} }} + b_{1} (\frac{1}{2})^{{1 + p_{1} }} \dot{e}_{0}^{{1 + 2p_{1} }} ) + k_{\sigma } e_{0} \\ = & e_{2} + \frac{\pi }{{p_{1} T_{s1} \sqrt {a_{1} b_{1} } }}(a_{1} (\frac{1}{2})^{{1 - p_{1} }} e_{1}^{{1 - 2p_{1} }} + b_{1} (\frac{1}{2})^{{1 + p_{1} }} e_{1}^{{1 + 2p_{1} }} ) + k_{\sigma } e_{0} \\ \end{aligned}$$

Among them the sliding surface gains are $a_{1} ,b_{1} ,k_{\sigma } > 0$, $0 < p_{1} < 1$, and $T_{s1} > 0$ is the predefined time.

### Theorem 2

*For any PDT* $T_{s1} > 0$, *when the sliding surface* $\sigma = 0$, *the position tracking error* $e_{1}$ *located within the sliding surface* Eq. (19) *will converge to zero within the PDT* $T_{s1}$.

### Proof

When the sliding surface is $\sigma = 0$, there is

20 $$\dot{e}_{1} = - \frac{\pi }{{p_{1} T_{s1} \sqrt {a_{1} b_{1} } }}(a_{1} (\frac{1}{2})^{{1 - p_{1} }} e_{1}^{{1 - 2p_{1} }} + b_{1} (\frac{1}{2})^{{1 + p_{1} }} e_{1}^{{1 + 2p_{1} }} ) - k_{\sigma } e_{0}$$

Construct the Lyapunov function $V_{0} = \frac{1}{2}e_{1}^{2}$ and take its derivative

21 $$\begin{aligned} \dot{V}_{0} = & e_{1}^{{}} \dot{e}_{1}^{{}} = - \frac{\pi }{{p_{1} T_{s1} \sqrt {a_{1} b_{1} } }}(a_{1} (\frac{1}{2})^{{1 - p_{1} }} e_{1}^{{2 - 2p_{1} }} + b_{1} (\frac{1}{2})^{{1 + p_{1} }} e_{1}^{{2 + 2p_{1} }} ) - k_{\sigma } e_{1}^{{}} e_{0} \\ = & - \frac{\pi }{{p_{1} T_{s1} \sqrt {a_{1} b_{1} } }}(a_{1} (\frac{1}{2})^{{1 - p_{1} }} e_{1}^{{2 - 2p_{1} }} + b_{1} (\frac{1}{2})^{{1 + p_{1} }} e_{1}^{{2 + 2p_{1} }} ) - k_{\sigma } \int_{0}^{t} {e_{1}^{2} (\tau )d\tau } \\ & \le - \frac{\pi }{{p_{1} T_{s1} \sqrt {a_{1} b_{1} } }}(a_{1} V_{0}^{{1 - p_{1} }} + b_{1} V_{0}^{{1 + p_{1} }} ) \end{aligned}$$

As it is described in Theorem 1, the position tracking error $e_{1}$ has the feature to converge to zero within the PDT $T_{s1}$. And then according to $e_{0} (t) = \int_{0}^{t} {e_{1} (\tau )d\tau }$ the fact that the SMS (15) is an integral sliding mode, when the seeker coordinator servo position tracking error is large, the increase of the integral term will damage the transient performance of the system, especially when there are constraints on the control input, which will generate the integral Windup effect and easily lead to unstable seeker coordinator servo position tracking. To address this issue, with reference to^51^ , it can be modified the sliding surface (19) to a nonlinear sliding surface as follows:

22 $$\left\{ \begin{gathered} \sigma = e_{2} + \frac{\pi }{{p_{1} T_{s1} \sqrt {a_{1} b_{1} } }}(a_{1} (\frac{1}{2})^{{1 - p_{1} }} e_{1}^{{1 - 2p_{1} }} + b_{1} (\frac{1}{2})^{{1 + p_{1} }} e_{1}^{{1 + 2p_{1} }} ) + k_{\sigma } \psi \hfill \\ \dot{\psi } = \left\{ \begin{gathered} \beta \sin \frac{{\pi e_{1} }}{2\beta }\;\;\;\;\;|e_{1} | < \beta \hfill \\ \;\;\;\;\beta \;\;\;\;\;\;\;\;\;\;\;e_{1} \ge \beta \hfill \\ \;\; - \beta \;\;\;\;\;\;\;\;\;\;\;e_{1} \le - \beta \hfill \\ \end{gathered} \right. \hfill \\ \end{gathered} \right.$$

Among them, $\beta$ is the parameter to be designed. This design can limit the small error method and large error amplitude in the integral term, and achieve ideal steady-state error when selecting different parameters $\beta$.Taking the derivative of the sliding surface $\sigma$ with respect to time $t$ can obtain.

23 $$\begin{aligned} \dot{\sigma } \, = \, & \dot{e}_{2} + \frac{\pi }{{p_{1} T_{s1} \sqrt {a_{1} b_{1} } }}(a_{1} (1 - 2p_{1} )(\frac{1}{2})^{{1 - p_{1} }} e_{1}^{{ - 2p_{1} }} + b_{1} (1 + 2p_{1} )(\frac{1}{2})^{{1 + p_{1} }} e_{1}^{{2p_{1} }} )\dot{e}_{1} + k_{\sigma } \dot{\psi } \\ \, = \, & M_{0} e_{2} + N_{0} v + G + M_{0} \dot{y}_{d} - \ddot{y}_{d} \\&\quad + \frac{\pi }{{p_{1} T_{s1} \sqrt {a_{1} b_{1} } }}(a_{1} (1 - 2p_{1} )(\frac{1}{2})^{{1 - p_{1} }} e_{1}^{{ - 2p_{1} }} + b_{1} (1 + 2p_{1} )(\frac{1}{2})^{{1 + p_{1} }} e_{1}^{{2p_{1} }} )e_{2} + k_{\sigma } g(e_{1} ) \end{aligned}$$

where $$g(e_{1} ) = \left\{ \begin{gathered} \beta \sin \frac{{\pi e_{1} }}{2\beta }\;\;\;\;\;|e_{1} | < \beta \hfill \\ \;\;\;\;\beta \;\;\;\;\;\;\;\;\;\;\;e_{1} \ge \beta \hfill \\ \;\; - \beta \;\;\;\;\;\;\;\;\;\;\;e_{1} \le - \beta \hfill \\ \end{gathered} \right.$$。

## Design of PDT sliding mode controller for CPS servo system

### Design of PTCSMAC without perturbations

In this section we have considered the perturbation and uncertainties in the system. When the system parameter perturbation is considered, the uncertainty term $G$ is simplified as $G_{1} = - N_{0} \theta + d(t)$. According to the boundedness of external interference $d(t)$ and the boundedness of control input bias, it can be known that the uncertainty term is bounded $G_{1}$, that is, $|G_{1} | = | - N_{0} \theta + d(t)| \le b_{u}$. Therefore, a PDT-SMC is designed for seeker coordinator servo systems without parameter perturbations.

Construct Lyapunov function $V_{1} = \frac{1}{2}\sigma^{2}$. Derivation of $V_{1}$ with respect to time $t$

24 $$\begin{aligned} \dot{V}_{1}& = \, \sigma \dot{\sigma } = \sigma (M_{0} e_{2} + N_{0} v + G_{1} + M_{0} \dot{y}_{d} - \ddot{y}_{d} \\ \quad +\, \frac{\pi }{{p_{1} T_{s1} \sqrt {a_{1} b_{1} } }}(a_{1} (1 - 2p_{1} )(\frac{1}{2})^{{1 - p_{1} }} e_{1}^{{ - 2p_{1} }} \\ \quad +\, b_{1} (1 + 2p_{1} )(\frac{1}{2})^{{1 + p_{1} }} e_{1}^{{2p_{1} }} )e_{2} + k_{\sigma } g(e_{1} )) \\ \end{aligned}$$

Design a PDT convergent sliding mode control without parameter perturbations as

25 $$\begin{aligned} v = & - \frac{1}{{N_{0} }}(M_{0} e_{2} + b_{u} sign(\sigma ) + M_{0} \dot{y}_{d} - \ddot{y}_{d} + \frac{\pi }{{p_{1} T_{s1} \sqrt {a_{1} b_{1} } }}(a_{1} (1 - 2p_{1} )(\frac{1}{2})^{{1 - p_{1} }} e_{1}^{{ - 2p_{1} }} \\ + b_{1} (1 + 2p_{1} )(\frac{1}{2})^{{1 + p_{1} }} e_{1}^{{2p_{1} }} )e_{2} + k_{\sigma } g(e_{1} )) \\ - \frac{\pi }{{p_{2} N_{0} T_{s2} \sqrt {a_{2} b_{2} } }}(a_{2} (\frac{1}{2})^{{1 - p_{2} }} \sigma^{{1 - 2p_{2} }} + b_{2} (\frac{1}{2})^{{1 + p_{2} }} \sigma^{{1 + 2p_{2} }} ) \\ \end{aligned}$$

Then one can get the following expression:

26 $$\begin{aligned} \dot{V}_{1} = & \sigma (G_{1} - d_{u} sign(\sigma )) - \frac{\pi }{{p_{2} T_{s2} \sqrt {a_{2} b_{2} } }}\sigma (a_{2} (\frac{1}{2})^{{1 - p_{2} }} \sigma^{{1 - 2p_{2} }} + b_{2} (\frac{1}{2})^{{1 + p_{2} }} \sigma^{{1 + 2p_{2} }} ) \\ \le & - \frac{\pi }{{p_{2} T_{s2} \sqrt {a_{2} b_{2} } }}(a_{2} V_{1}^{{1 - p_{2} }} + b_{2} V_{1}^{{1 + p_{2} }} ) \\ \end{aligned}$$

According to Theorem 1, the SMS $\sigma$ should satisfies the convergence and approaches to zero within the PDT $T_{s2}$. It is obvious that the moment system state reaches and stabilizes within the SMS(15) (i.e. $\sigma = 0$), the position tracking error $e_{1} (t)$ will converge to the origin within the PDT $T_{s1}$, ultimately achieving the convergence of the position tracking error $e_{1} (t)$ to zero within $T_{s} = T_{s1} + T_{s2}$.

#### Theorem 3

*When parameter perturbations in the seeker coordinator servo system* Eq. (7) *are not considered, for any PDT* $T_{s1} > 0,$, $T_{s2} > 0$, *for which the adaptive control law is*:

27 $$u(t) = v(t) - \theta (t)$$

*where* $v(t)$ *is the overall control law that can be described as follows*:

28 $$\begin{aligned} v = & - \frac{1}{{N_{0} }}(M_{0} e_{2} + b_{u} sign(\sigma ) + M_{0} \dot{y}_{d} - \ddot{y}_{d} + \frac{\pi }{{p_{1} T_{s1} \sqrt {a_{1} b_{1} } }}(a_{1} (1 - 2p_{1} )(\frac{1}{2})^{{1 - p_{1} }} e_{1}^{{ - 2p_{1} }} \\ + b_{1} (1 + 2p_{1} )(\frac{1}{2})^{{1 + p_{1} }} e_{1}^{{2p_{1} }} )e_{2} + k_{\sigma } g(e_{1} )) \\ - \frac{\pi }{{p_{2} N_{0} T_{s2} \sqrt {a_{2} b_{2} } }}(a_{2} (\frac{1}{2})^{{1 - p_{2} }} \sigma^{{1 - 2p_{2} }} + b_{2} (\frac{1}{2})^{{1 + p_{2} }} \sigma^{{1 + 2p_{2} }} ) \\ \end{aligned}$$

29 $$\theta (t) = \left\{ \begin{gathered} 0\;\;\;\;\;\;\;\;\;\;\;\;\;\;\;\;\;\;\;\;\;\;|v| < u_{{\text{M}}} \hfill \\ v - {\text{sign}} (v)u_{{\text{M}}} \;\;\;\;\;|v| \ge u_{{\text{M}}} \hfill \\ \end{gathered} \right.$$

30 $$\left\{ \begin{gathered} \sigma = e_{2} + \frac{\pi }{{p_{1} T_{s1} \sqrt {a_{1} b_{1} } }}(a_{1} (\frac{1}{2})^{{1 - p_{1} }} e_{1}^{{1 - 2p_{1} }} + b_{1} (\frac{1}{2})^{{1 + p_{1} }} e_{1}^{{1 + 2p_{1} }} ) + k_{\sigma } \psi \hfill \\ \dot{\psi } = \left\{ \begin{gathered} \beta \sin \frac{{\pi e_{1} }}{2\beta }\;\;\;\;\;|e_{1} | < \beta \hfill \\ \;\;\;\;\beta \;\;\;\;\;\;\;\;\;\;\;e_{1} \ge \beta \hfill \\ \;\; - \beta \;\;\;\;\;\;\;\;\;\;\;e_{1} \le - \beta \hfill \\ \end{gathered} \right. \hfill \\ \end{gathered} \right.$$

*It is hence proved that the CPS seeker coordinator servo is globally stable at the predefined time*, *and the position tracking error* $e_{1}$ *obviously has the convergence within the PDT* $T_{s} = T_{s1} + T_{s2}$.

### Design of PTCSMAC with all perturbations, input control attacks and disturbances

In controller Eq. (22), it is necessary to obtain the upper bound $G_{1}$ information of external interference $b_{u}$ in advance, and the controller does not consider the parameter perturbation of the CPS seeker servo system. To improve the practical engineering application of the designed controller and minimize the use of motor information and simplify the structure of the controller, a model free sliding mode adaptive controller considering motor parameter perturbations will be established below.

When considering motor parameter perturbation, $G = \Delta Mx_{2} - N_{0} \theta + \Delta N(v - \theta ) + d(t)$, then the derivative of the Lyapunov function $V_{1}$ is

31 $$\begin{aligned} \dot{V}_{1} & = \sigma (M_{0} e_{2} + N_{0} v + G + M_{0} \dot{y}_{d} - \ddot{y}_{d} \\ & +\, \frac{\pi }{{p_{1} T_{s1} \sqrt {a_{1} b_{1} } }}(a_{1} (1 - 2p_{1} )(\frac{1}{2})^{{1 - p_{1} }} e_{1}^{{ - 2p_{1} }} \\ &+\, b_{1} (1 + 2p_{1} )(\frac{1}{2})^{{1 + p_{1} }} e_{1}^{{2p_{1} }} )e_{2} + k_{\sigma } g(e_{1} )) \\ & \sigma (N_{0} v + F - \ddot{y}_{d} ) \\ \end{aligned}$$

where: $F = M_{0} e_{2} + G + M_{0} \dot{y}_{d} + \frac{\pi }{{p_{1} T_{s1} \sqrt {a_{1} b_{1} } }}(\frac{{a_{1} (1 - 2p_{1} )}}{{2^{{1 - p_{1} }} }}e_{1}^{{ - 2p_{1} }} + b_{1} \frac{{(1 + 2p_{1} )}}{{2^{{1 + p_{1} }} }}e_{1}^{{2p_{1} }} )e_{2} + k_{\sigma } g(e_{1} )$ is the system packaging part, that has approximated by Extreme learning machine (ELM), and $\hat{F}$ is the estimated value of $F$ Extreme learning machine. Then a PDT convergent controller based on ELM can be designed:

32 $$v = - \frac{1}{{N_{0} }}(\hat{F} - \ddot{y}_{d} ) - \frac{\pi }{{p_{2} N_{0} T_{s2} \sqrt {a_{2} b_{2} } }}(a_{2} (\frac{1}{2})^{{1 - p_{2} }} \sigma^{{1 - 2p_{2} }} + b_{2} (\frac{1}{2})^{{1 + p_{2} }} \sigma^{{1 + 2p_{2} }} )$$

After substituting Eq. (32) into Eq. (31), there is

33 $$\begin{aligned} \dot{V}_{1} \, = \,& \sigma (F - \hat{F}) - \frac{\pi }{{p_{2} T_{s2} \sqrt {a_{2} b_{2} } }}(a_{2} (\frac{1}{2})^{{1 - p_{2} }} \sigma^{{2 - 2p_{2} }} + b_{2} (\frac{1}{2})^{{1 + p_{2} }} \sigma^{{2 + 2p_{2} }} ) \\ & =\, \sigma (F - \hat{F}) - \frac{\pi }{{p_{2} T_{s2} \sqrt {a_{2} b_{2} } }}(a_{2} V_{1}^{{1 - p_{2} }} + b_{2} V_{1}^{{1 + p_{2} }} ) \\ \end{aligned}$$

When the Extreme learning machine accurately estimates the $F$ of the packaging part, there is $\dot{V}_{1} \le - \frac{\pi }{{p_{2} T_{s2} \sqrt {a_{2} b_{2} } }}(a_{2} V_{1}^{{1 - p_{2} }} + b_{2} V_{1}^{{1 + p_{2} }} )$, which means that the sliding surface will converge to zero within the PDT $T_{s2}$. When the SMS is $\sigma (t) = 0$, the position error $e_{1} (t)$ located within that SMS which signifies that it will converges to zero within $T_{s1}$. It implies that respective error $e_{1} (t)$ of the CPS seeker servo system has clear convergence within the PDT $T_{s} = T_{s1} + T_{s2}$.

Next, use the ELM to approximate the encapsulated term $F$. The Extreme learning machine^52,53^ uses a single hidden layer Feedforward neural network with $\tilde{N}$ nodes, the Activation function is $h({\mathbf{x}})$, and the mathematical description of the extreme learning machine approaching that encapsulated term $F$ is as follows:

34 $$\sum\limits_{i = 1}^{{\tilde{N}}} {{\mathbf{w}}_{i} h_{i} ({\mathbf{c}}_{i} \cdot {\mathbf{z}},\delta_{i} )} = F$$

where $z = [e_{1} ,e_{2} ]^{\rm T}$ is the input information of ELM, ${\mathbf{c}}_{i} = [c_{i1} ,c_{i2} ]^{\rm T}$ represents connection weight of the $i$ hidden layer and the input nodes, $\delta_{i}$ specifies the threshold, $w_{i}$ denotes the weight of the $i$ hidden and output nodes respectively. While ${\mathbf{c}}_{i} \cdot {\mathbf{z}}_{j}$ characterizes the inner product of vector ${\mathbf{c}}_{i}$ and vector ${\mathbf{z}}_{j}$. In ELM, the connection weight ${\mathbf{c}}_{i}$ and node threshold $\delta_{i}$ are randomly generated^54,55^ and are external weight vectors to be identified through continuous learning and training, ELM can find the optimal weight vector $w_{i}$ to approximate the encapsulation term $w^{*}$, i.e.

35 $$F = \sum\limits_{i = 1}^{{\tilde{N}}} {w_{i}^{*} h_{i} (c_{i} z,\delta_{i} )} = h^{\rm T} (z)w_{{}}^{*}$$

Among them, $h(z) = (h_{1} (c_{1} z,\delta_{1} ), \cdots ,h_{{\tilde{N}}} (c_{{\tilde{N}}} z,\delta_{{\tilde{N}}} ))^{\rm T}$. The optimal weight ${\mathbf{w}}^{*}$ of ELM cannot be directly obtained in practical engineering applications^56,57^, ELM cannot accurately approximate $F$ and can only obtain an estimate of $F$ which is given as follows.

36 $$\hat{F} = \sum\limits_{i = 1}^{{\tilde{N}}} {\hat{w}_{i}^{{}} h_{i} (c_{i} z,\delta_{i} )} = h^{\rm T} (z)\hat{w}$$

where $\hat{w}_{i}^{{}}$ is the estimation of the optimal value $w_{i}^{*}$. Note $\tilde{w} = w^{*} - \hat{w}$ and construct the Lyapunov function $V_{2} = V_{1} + \frac{1}{2\gamma }\tilde{w}^{\rm T} \tilde{w}$, then

$$\begin{aligned} \dot{V}_{2} = & \sigma (F - \hat{F}) - \frac{\pi }{{p_{2} T_{s2} \sqrt {a_{2} b_{2} } }}(a_{2} (\frac{1}{2})^{{1 - p_{2} }} \sigma^{{2 - 2p_{2} }} + b_{2} (\frac{1}{2})^{{1 + p_{2} }} \sigma^{{2 + 2p_{2} }} ) - \frac{1}{\gamma }\tilde{w}^{\rm T} \dot{\hat{w}} \\ = & - \frac{\pi }{{p_{2} T_{s2} \sqrt {a_{2} b_{2} } }}(a_{2} V_{1}^{{1 - p_{2} }} + b_{2} V_{1}^{{1 + p_{2} }} ) + \sigma h^{\rm T} (z)\tilde{w} - \frac{1}{\gamma }\tilde{w}^{\rm T} \dot{\hat{w}} \\ = & - \frac{\pi }{{p_{2} T_{s2} \sqrt {a_{2} b_{2} } }}(a_{2} V_{1}^{{1 - p_{2} }} + b_{2} V_{1}^{{1 + p_{2} }} ) + \tilde{w}^{\rm T} (h(z)\sigma - \frac{1}{\gamma }\dot{\hat{w}}) \\ \end{aligned}$$

When the outer weight of ELM is

37 $$\dot{\hat{w}} = \gamma h(z)\sigma$$

then

$$\dot{V}_{2} \le - \frac{\pi }{{2p_{2} T_{s2} \sqrt {a_{2} b_{2} } }}(a_{2} V_{2}^{{1 - p_{2} }} + b_{2} V_{2}^{{1 + p_{2} }} )$$

The above equation indicates that the seeker coordinator servo system is globally PDT stable, and the SMS $\sigma$ will converge to zero within the PDT $T_{s2}$.

#### Theorem 4

*It is now confirmed that within the PDT* $$T_{s1} > 0,$$$$T_{s2} > 0$$, *the controller is guaranteed to stable and control law is given as follows*:

38 $$u(t) = v(t) - \theta (t)$$

*where the overall adaptive control law can be described as follows*:

39 $$v = - \frac{1}{{N_{0} }}(\hat{F} - \ddot{y}_{d} ) - \frac{\pi }{{p_{2} N_{0} T_{s2} \sqrt {a_{2} b_{2} } }}(a_{2} (\frac{1}{2})^{{1 - p_{2} }} \sigma^{{1 - 2p_{2} }} + b_{2} (\frac{1}{2})^{{1 + p_{2} }} \sigma^{{1 + 2p_{2} }} )$$

40 $$\theta (t) = \left\{ \begin{gathered} 0\;\;\;\;\;\;\;\;\;\;\;\;\;\;\;\;\;\;\;\;\;\;|v| < u_{{\text{M}}} \hfill \\ v - {\text{sign}} (v)u_{{\text{M}}} \;\;\;\;\;|v| \ge u_{{\text{M}}} \hfill \\ \end{gathered} \right.$$

41 $$\left\{ \begin{gathered} \sigma = e_{2} + \frac{\pi }{{p_{1} T_{s1} \sqrt {a_{1} b_{1} } }}(a_{1} (\frac{1}{2})^{{1 - p_{1} }} e_{1}^{{1 - 2p_{1} }} + b_{1} (\frac{1}{2})^{{1 + p_{1} }} e_{1}^{{1 + 2p_{1} }} ) + k_{\sigma } \psi \hfill \\ \dot{\psi } = \left\{ \begin{gathered} \beta \sin \frac{{\pi e_{1} }}{2\beta }\;\;\;\;\;|e_{1} | < \beta \hfill \\ \;\;\;\;\beta \;\;\;\;\;\;\;\;\;\;\;e_{1} \ge \beta \hfill \\ \;\; - \beta \;\;\;\;\;\;\;\;\;\;\;e_{1} \le - \beta \hfill \\ \end{gathered} \right. \hfill \\ \end{gathered} \right.$$

42 $$\hat{F} = h^{\rm T} (z)\hat{w}$$

43 $$\dot{\hat{w}} = \gamma h(z)\sigma$$

The seeker coordinator servo system (Eq. 11) is stable for the PDT, and the error $e_{1}$ has obvious convergence within the predefined interval $T_{s} = T_{s1} + T_{s2}$.

Our designed control has the following intrinsic robustness and adaptability features:

- The proposed control system has the capacity to function well without requiring complex model knowledge is one of its unique characteristics. In situations when the precise model features are unclear or constantly changing, this guarantees that the system will continue to function effectively.
- The nonsingular design of our proposed controller eliminates risks of singularity issues in control systems. This guarantees efficient, faultless operations.
- The proposed PTCSMAC control may be deployed and operated with more freedom since its performance is not dependent on a particular initial condition.
- The system is flexible and adaptive to a variety of cyber threats and operational circumstances since it is not substantially dependent on any particular control parameters for its operations and efficiency.

#### Remark

According to Theorem 4, the PDT convergent SMC based on Extreme learning machine does not contain the model information of seeker coordinator servo. Therefore, this controller is a model free controller and is applicable to the control of any seeker coordinator servo system. At the same time, in this controller, the derivative information of the SMS is not required, so that the controller does not have singularity problems.

## Simulation results and analysis

**Figure 3:**
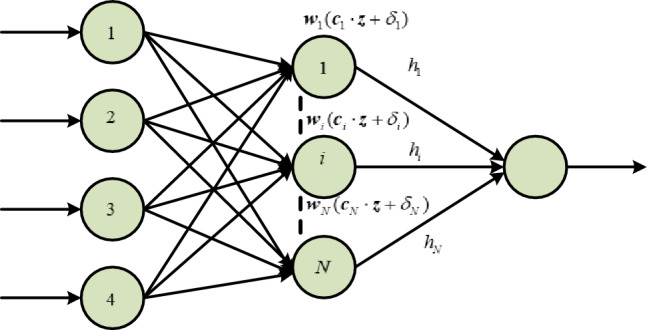
ELM basic structure diagram.

**Figure 4:**
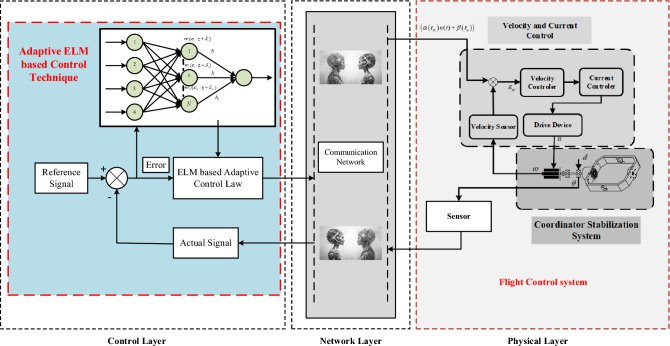
Overall control structure of the proposed PTCSMAC.

We are going to present and analyze the results of our simulation experiments in this Section, with an emphasis on the effectiveness of the suggested PTCSMAC in the context of a seeker coordinator CPS servo system. First, as shown in Fig. 3, the demonstration of ELM and then we can use that to obtain the PTCSMAC's general control structure. After then, the conversation will shift to a thorough analysis of the system's parameters and how they interact with the requirements of the suggested controller to affect overall performance. Overall control CPS servo position control system is sown in Fig. 4.

This section also aims to validate the PTCSMAC's performance across different operational conditions, ensuring that it meets the high-precision control demands of the CPS seeker servo system, particularly its response to the reference position signal and resilience to different level of cyber-attacks as well as potential disturbances. The practical Seeker Coordinator Servo System Parameters are demonstrated in the following Table 1.

**Table 1:** Seeker coordinator servo system parameters.

Parameter description	Symbol (unit)	Numerical value
Electric torque coefficient	*K*_*T*_	0.0433
Back electromotive force coefficient	*K*_e_	0.025
Load torque of the detection system	*T*_*L*_/(N∙m)	0.01
Motor’s moment of inertia	*J*/(kg∙m^2^)	0.0000293
Coefficient of viscosity	*B*_*m*_	0.001088
Reduction ratio of transmission mechanism	*ηg*	5
Friction torque	*kv*	0.001088
	*fc*	0.00001
	*fs*	0.00002
	${\dot{{\varvec{\theta}}}}_{{\varvec{s}}}$	1
Ripple torque	*A*_*R*_	0.0002

We have taken the following parameters for the controller settings as depicted in Table 2 and these gains as well as settings will remains unchanged in all the cases as discussed below. Some of the parameters such as predefined time and activation function has been changed according to simulation experiment in order to analyze the effectiveness of our proposed control technique (PTCSMAC).

**Table 2:** Proposed PTCSMAC controller parameters.

Element	Description
Simulation total time	t = 10 s
Initial value	$x_{1} (0) = 0.5,x_{2} (0) = 0.5,\hat{\theta }(0) = 0.5$
Upper bound of control input	$u_{M}$ = 30
Predefined convergence time	$T_{s} = T_{s1} + T_{s2}$
Sliding mode surface	${\text{a}}_{1} { = }10,b_{1} = 11.5,p_{1} = 0.3,T_{s1} = 1$
Sliding mode adaptive controller	$k_{\sigma }$ = 1.1,$T_{s2} = 1{\text{s}}$,${\text{a}}_{2} { = }10,b_{2} = 11.5$;$\gamma$ = 5,$p_{2} = 0.3,T_{s2} = 1$
ELM (activation function)	$h_{i} (z) = \frac{1}{{1 + e^{{ - {\mathbf{zc}}_{i} + b_{i} }} }}$
Reference position signal	$x_{r} = \sin (t)$

### Case 1: attack free (attack free scenario)

In this section, we have analyzed and explained the simulation results, which demonstrate the effectiveness of our proposed control technique (PTCSMAC), in the context of a normal case when cyberattack does not occur. Furthermore, we have also covered these aspects: position tracking accuracy, tracking error and control effort of our proposed control technique (PTCSMAC).

$${\text{Case1}}:{\text{ attack free scenario}}\left\{ \begin{gathered} \alpha \left( {t_{m} } \right) = 1 \hfill \\ \beta (t_{a} ) = 0 \hfill \\ \end{gathered} \right.$$

**Figure 5:**
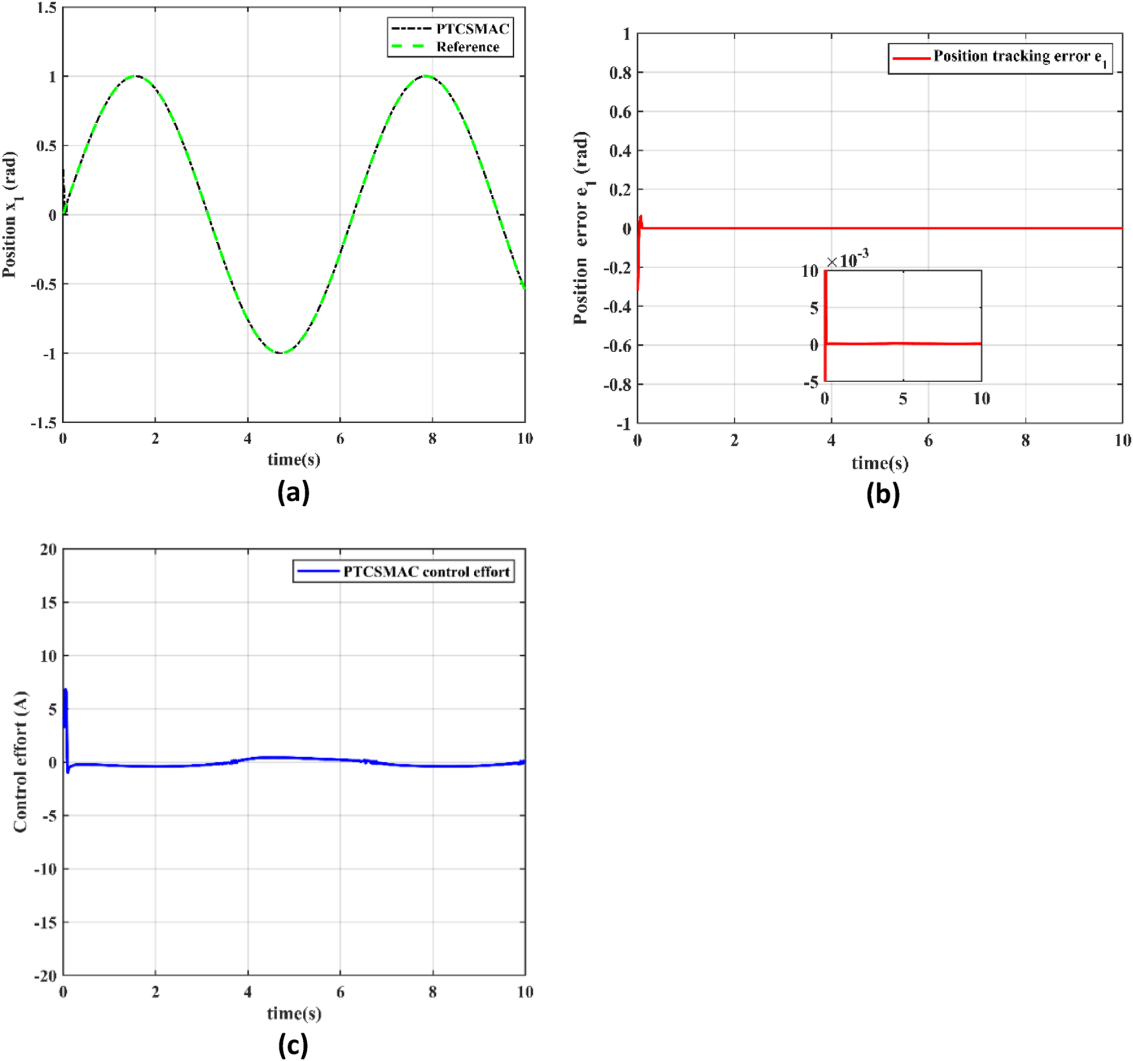
Proposed control tracking performance. (**a**) Reference position v/s output position trajectory tracking (black line is our proposed PTCSMAC control while the red line is given reference). (**b**) Position tracking error (red line is error under our proposed PTCSMAC. (**c**) Control effort of our proposed PTCSMAC.

The performance of the proposed PTCSMAC control technique in an attack-free case is thoroughly evaluated in Fig. 5, which is represented by three subfigures that each represent a distinct feature of system behavior.

Figure 5a illustrates the tracking between reference position trajectory and the output position trajectory, where the red line signifies the given reference trajectory and the black line represents the system's response when under our proposed PTCSMAC control. The position tracking suggests that our proposed PTCSMAC control law can achieve perfect tracking, which precisely follows the given reference position. Figure 5b demonstrates the error of the position tracking. The red line, which shows the error under our proposed control (PTCSMAC), is seen to be quite small and goes up to the micro level, highlighting the control technique's accuracy. For systems that require precise control, a small error margin indicates that the system can track the reference signal very precisely and with a high degree of accuracy. Figure 5c, deals with the control effort that the PTCSMAC demands. Our proposed PTCSMAC appears to function effectively without requiring significant or abrupt changes in control signals, based on the representation of a steady and smooth control effort. This type of smooth control effort is a sign of a reliable control system that can continue operating without straining the actuators or using too much energy. Our proposed PTCSMAC control shows an excellent performance with perfect tracking, minimum error, and a smooth control effort in Case 1, which is free from any attacks, highlighting its effectiveness and reliability under ideal conditions.

### Case 2: low level attack

In this section, we are going to analyze and explain the research results, which compares the effectiveness of three control techniques, including our proposed novel approach (PTCSMAC), in the context of a low-level cyberattack. This analysis has covered three comparative aspects: position tracking accuracy, tracking error and control effort.

$$\left\{ \begin{gathered} \left. \begin{gathered} \alpha \left( {t_{m} } \right) = 1 \hfill \\ \beta (t_{a} ) = 0 \hfill \\ \end{gathered} \right\}\,\,\,,\,\,\,\,\,\,\,\,\,\,\,\,\,\,\,\,\,\,\,\,\,\,\,\,\,\,\,\,\,\,\,\,\,\,\,\,\,\,\,t < 2 \hfill \\ \left. \begin{gathered} \alpha \left( {t_{m} } \right) = 0.5 + 0.5.e^{(0.1t)} \hfill \\ \beta (t_{a} ) = 0.5\cos^{2} \left( {x_{2} } \right) \hfill \\ \end{gathered} \right\}\,,\,\,\,2 \le t \le 5 \hfill \\ \left. \begin{gathered} \alpha \left( {t_{m} } \right) = 0.03 + 0.01.e^{(0.1t)} \hfill \\ \beta (t_{a} ) = 0.1\cos^{2} \left( {x_{2} } \right) \hfill \\ \end{gathered} \right\},\,\,\,other\, \hfill \\ \end{gathered} \right.$$

**Figure 6:**
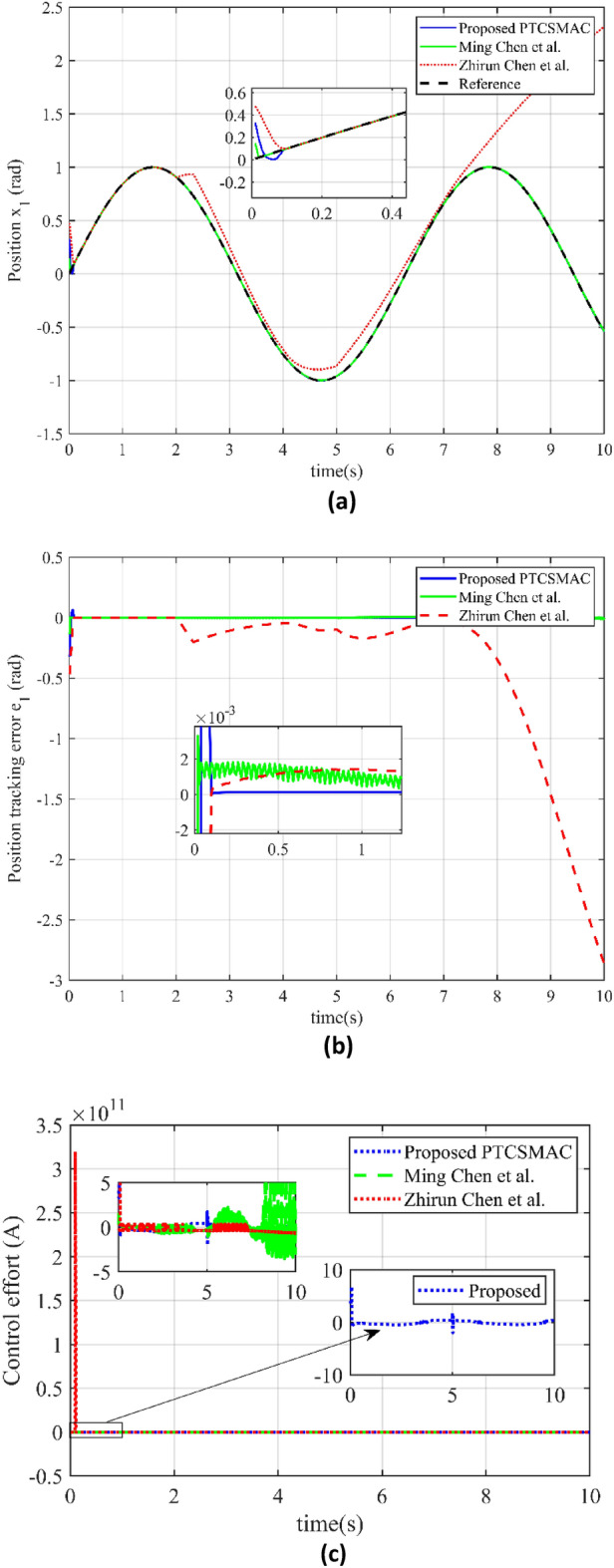
Comparison of tracking and control performance in case 2. (**a**) Reference position v/s output position trajectory tracking (blue line is our proposed PTCSMAC control tracking, red line is the tracking of Ref^39^, while green line is the tracking of Ref^37^). (**b**) Position tracking error comparison (blue line is our proposed PTCSMAC, red line is the tracking error of Ref^39^, while green line is the tracking error of Ref^37^). (**c**) Control effort comparison of different control laws (blue line is our proposed PTCSMAC control law, red line is the control effort of Ref^39^, while green line is the control effort of Ref^37^).

For case 2 and Fig. 6 have thoroughly examined the performance of three control techniques in terms of position tracking accuracy and control effort, particularly under conditions of multiple uncertainties and cyberattacks as well as shows the tracking errors. In this study, Fig. 6a shows the resilience of the proposed control system under challenging conditions by comparing the performance of three different control strategies under low-level attacks (Case2). The newly proposed predefined time convergence sliding mode adaptive controller (PTCSMAC), shown as a blue line on the graph, is the main subject of discussion. This line shows the system's output location, which is influenced by several uncertainties and malicious attacks, but nevertheless closely tracks the reference position trajectory. PTCSMAC's performance is compared to two other control approaches found in the literature: Ming Chen et al.'s method (green line) and Zhirun Chen et al.'s method (red line). It is demonstrated that the tracking performance of Ming Chen et al.'s method is not optimal, making it inadequate for precision control. However, under the identical conditions, Zhirun Chen et al.'s control technique encounters severe singularity problems, which results in a complete failure to track the target trajectory. In conclusion, Fig. 6a clearly shows how the PTCSMAC works better than the approaches which have been compared, maintaining precision and reliability in position tracking even in the case of a system compromise, making it a notably advantageous approach in the face of challenges like cyberattacks and uncertainty.

Figure 6b offers a comparison of position tracking errors. The blue line represents the error for the proposed PTCSMAC, showing significantly smaller deviations from the desired position, which suggests a high level of precision in tracking. While the red and green lines represent the tracking errors for the control techniques by Zhirun Chen et al. and Ming Chen et al., respectively. Zhirun Chen et al.'s method, as indicated by the red line, has pronounced tracking errors due to singularity issues that arise amongst uncertainties and cyberattacks, rendering it ineffective. Ming Chen et al.'s method, although fair in position tracking, is depicted by the green line and is characterized by a large tracking error, which reduces its reliability for precise control.

It can be seen from Fig. 6c which depicts the control effort exerted by the different control laws. The blue line for the PTCSMAC indicates a controlled and stable effort, which implies efficiency and reliability without excessive demand on system resources. In contrast, Ming Chen et al.'s method (green line) shows a high level of chattering in the control input, a phenomenon that can lead to premature wear and tear or damage to the actuators within the control system. As compared to the technique by Zhirun Chen et al. (red line), the control effort fails when it faces the cyberattacks due to the same singularity issues that affect its tracking ability, which could lead to system instability or failure. As we can see from Fig. 6c which highlights the superiority of our proposed PTCSMAC, that not only maintains a high reliability in tracking the desired trajectory but also operates with an efficient control effort. In a contrast with the other methods that struggle with large errors, system inefficiencies, and potential damage to the control system components, especially in the face of cyber-attacks and uncertainties.

### Case 3: high level attack

In this section, similar to above case 2, we are also analyzing and describe the rigorous results, in which we will compare the efficacy of different proposed control laws in different existing literature, with our proposed approach (PTCSMAC). In this section we will discuss worst case scenario the high-level cyberattack. Similarly, we will cover three comparative aspects: position tracking accuracy, tracking error and control effort in this worst case.

$${\text{Case3}}:{\text{ The worst-case scenario}}\left\{ \begin{gathered} \left. \begin{gathered} \alpha \left( {t_{m} } \right) = 1 \hfill \\ \beta (t_{a} ) = 0 \hfill \\ \end{gathered} \right\}\,\,\,,\,\,\,\,\,\,\,\,\,\,\,\,\,\,\,\,\,\,\,\,\,\,\,\,\,\,\,\,\,\,\,\,\,\,\,\,\,\,\,t < 2 \hfill \\ \left. \begin{gathered} \alpha \left( {t_{m} } \right) = 5 + 10.e^{(0.1t)} \hfill \\ \beta (t_{a} ) = 10.\cos^{2} \left( {x_{2} } \right) \hfill \\ \end{gathered} \right\}\,,\,\,\,2 \le t \le 5 \hfill \\ \left. \begin{gathered} \alpha \left( {t_{m} } \right) = 1 \hfill \\ \beta (t_{a} ) = 0 \hfill \\ \end{gathered} \right\}\,\,\,,\,\,\,other\, \hfill \\ \end{gathered} \right.$$

**Figure 7:**
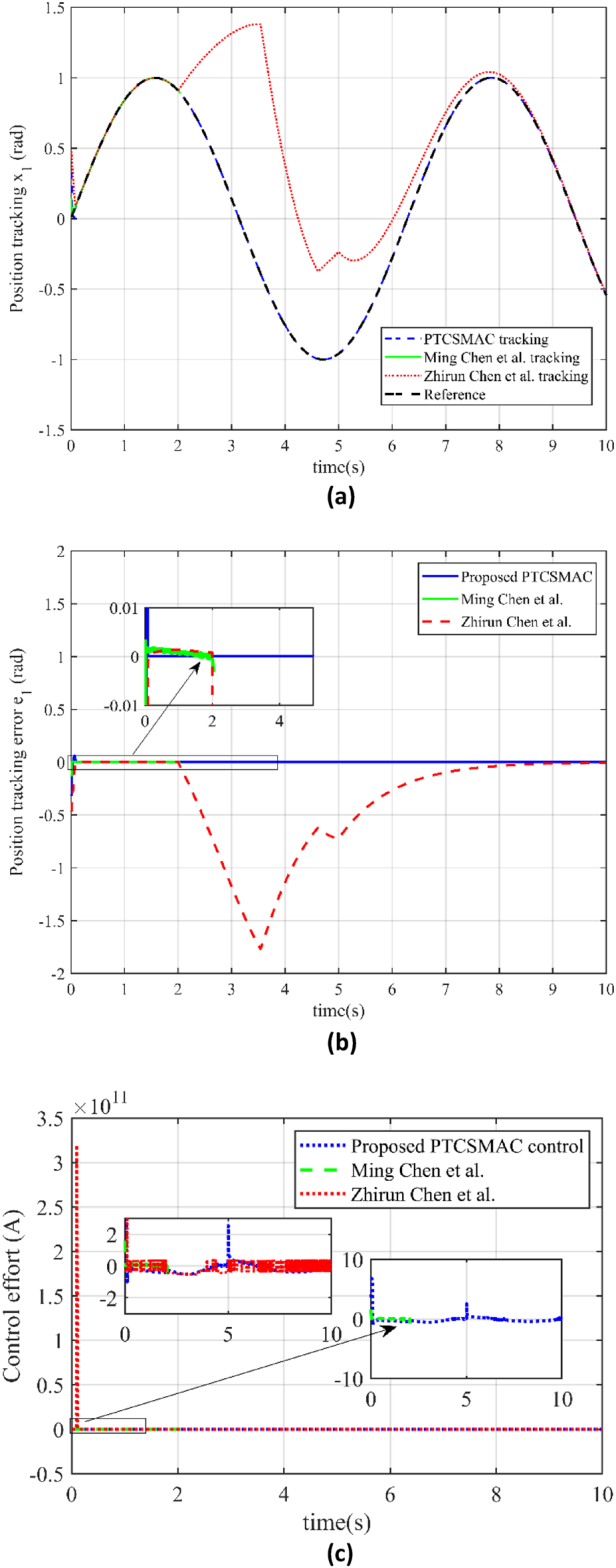
Comparison of tracking and control performance in case 3. (**a**) Case3: reference trajectory tracking (blue line is our proposed PTCSMAC control tracking, red line is the tracking of Ref^39^, while green line is the tracking of Ref^37^). (**b**) Case3: position tracking error comparison (blue line is our proposed PTCSMAC, red line is the tracking error of Ref^39^, while green line is the tracking error of Ref^37^). (**c**) Case3: control effort comparison of different control laws (blue line is our proposed PTCSMAC control law, red line is the control effort of Ref^39^, while green line is the control effort of Ref^37^).

In Fig. 7, we have investigated the performance of three different control techniques under a high-level cyber-attack scenario, offering a detailed assessment of their robustness and practicality in maintaining system control. Figure 7a illustrates position trajectory tracking is shown for the three different control including our proposed control law. The blue line, representing the proposed PTCSMAC control's tracking performance, shows a close adherence to the desired trajectory despite the high-level cyber-attack, indicating the controller's robustness. In stark contrast, the red and green lines, which represent the tracking by control techniques from references^39^ and^37^ respectively, deviate significantly from the reference trajectory. It shows the failure to maintain proper tracking under the high-level cyber-attack (case 3) conditions, with Ming Chen et al.'s method (green line) suffering from singularity in its control law, leading to an inability to follow the trajectory tracking at all. In Fig. 7b we have presented a comparison of the position tracking errors. Our proposed PTCSMAC's tracking error, depicted by the blue line, remains very small, which signifies its precision and reliability even when the system is under high-level cyber-attack. The red and green lines illustrate the errors for the methods by references^39^ and^37^, respectively. Both of these methods exhibit very large errors due to their failure to compensate for the disturbances introduced by the high-level cyber-attack, rendering them ineffective in this scenario. We also have compared the control effort required by the different control laws in Fig. 7c. Our proposed PTCSMAC's control effort under the high-level cyber-attack (case 3) conditions, shown by the blue line, indicates a smooth and consistent application of control, suggesting that the PTCSMAC can counteract the cyber-attack effects without demanding excessive energy or causing erratic system behavior. In comparison, the control efforts by references^37,39^, shown by the red and green lines, are likely erratic or inadequate, as these controls fail post-attack due to the same issues that affect their tracking ability. We can see that under a high-level cyber-attack, the PTCSMAC distinctly outperforms the compared methods by maintaining accurate trajectory tracking with minimal error and a smooth control effort. It shows that our proposed PTCSMAC's control has strong robustness and ability to withstand cyber-attacks and multiple uncertainties within the system, as distinct to the other referenced methods which fail to maintain control due to singularity issues.

### Predefined time impact on the system resilience against cyberattacks

We will analyze the seeker servo system's performance utilizing our PTCSMAC control over a range of specified times, *Ts* = 1 s, *Ts* = 1.2 s, *Ts* = 2 s, and *Ts* = 3 s (see Table 3), in this section. Three different scenarios will be used to conduct this evaluation: Case 1, which will be an attack-free case; Case 2, which will be a low-level cyberattack; and Case 3, which will be a high-level cyberattack. Our goal is to measure the tracking inaccuracy in every scenario and evaluate the accuracy and dependability of the PTCSMAC's position tracking capability. The study will concentrate on how the various predefined time impact the system's resilience to cyberattacks and tracking accuracy, offering valuable insights into the best configurations for servo position control under varying threat scenarios.

**Table 3:** Predefined convergence time setting.

Total predefined time	Parameters settings
$T_{s} = 1$	$T_{s1} = 0.5,T_{s2} = 0.5$
$T_{s} = 1.2$	$T_{s1} = 0.2,T_{s2} = 1$
$T_{s} = 2$	$T_{s1} = 1,T_{s2} = 1$
$T_{s} = 3$	$T_{s1} = 1,T_{s2} = 2$

**Figure 8:**
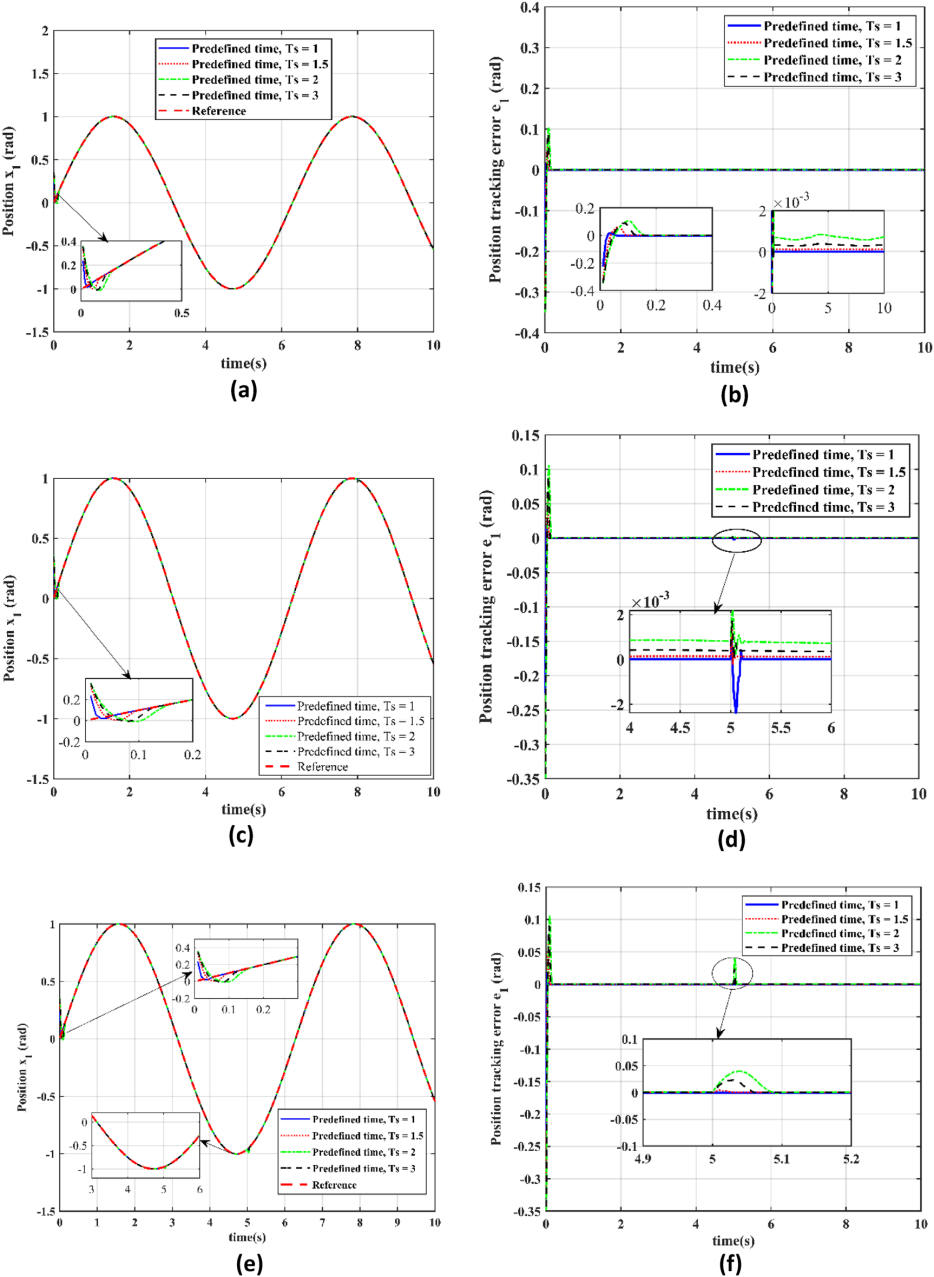
Performance evaluation at different predefined time settings for individual case. (**a**) Reference v/s output position trajectory tracking for different predefined time (*Ts*) in case 1. (**b**) Position tracking error for the different predefined time (*Ts*) in Case 1. (**c**) Reference v/s output position trajectory tracking for the different predefined time (*Ts*) in case 2. (**d**) Position tracking error for the different predefined time (*Ts*) in Case 2. (**e**) Reference trajectory position tracking for the different predefined time (*Ts*) in case 3. (**f**) Position tracking error for the different predefined time (*Ts*) in Case 3.

In this section we have analyzed the performance of a seeker servo system utilizing a predefined time convergence sliding mode adaptive controller (PTCSMAC) with various predefined time settings *Ts* = 1 s, *Ts* = 1.2 s, *Ts* = 2 s, and *Ts* = 3 s across three different cases, each representing varying levels of cyber threat. In Case 1 (attack-free scenario), the results for different *Ts* values are analyzed in terms of reference versus output position trajectory tracking Fig. 8a. As it can be seen from Fig. 8b the error perfectly converges up to micro level for all the predefined time. The PTCSMAC tracking of the position are shown in Fig. 8c as well as Fig. 8e to achieve perfect tracking across all predefined times, with the associated tracking error remaining minimal, which is crucial for high-precision control. Furthermore, in Case 2, which involves a low-level cyber-attack, the system's resilience is tested. Despite the adversarial conditions, the tracking Fig. 8c remains accurate for all predefined times, and the tracking error in Fig. 8d is consistently small, demonstrating the control's effectiveness. And for the Case 3, under a high-level cyber-attack, the robustness of the PTCSMAC is put to the eventual assessment. Under this most challenging scenario, our proposed controller maintains excellent trajectory tracking as depicted in Fig. 8e for all predefined time (*Ts*) values, and the position tracking error Fig. 8f continues to be negligible. We can analyze that our proposed control law PTCSMAC shows a remarkable capability to ensure precise and accurate position tracking of the seeker servo system, regardless of cyber-attack severity or predefined time settings. The small tracking errors observed in all cases underline the controller's stability for applications that demand high precision in servo position control under severe cyber-attacks and uncertainties.

### Control effort for the different cases with dissimilar predefined time settings

In this section our objective is to evaluate the control effort required by the PTCSMAC control law in three different cyber-attack scenarios at different specified time settings (*Ts* = 1 s,* Ts* = 1.2 s, *Ts* = 2 s, and Ts = 3 s) will be covered in the next section. Case 1 is the absence of an attack, Case 2 is a low-level attack, and Case 3 is a high-level attack. The goal is to evaluate how well and consistently the control effort performs under each preset time setting, making sure that the system runs without producing any negative chatter in any scenario. This fast control response is critical to the servo system's robustness and reliability. The main objective of the analysis is to show that even in the most extreme attack scenarios, the PTCSMAC control law can sustain a consistent and efficient control effort that is resistant to the seriousness of cyberthreats, preserving system performance and stability without the negative consequences of chattering.

**Figure 9:**
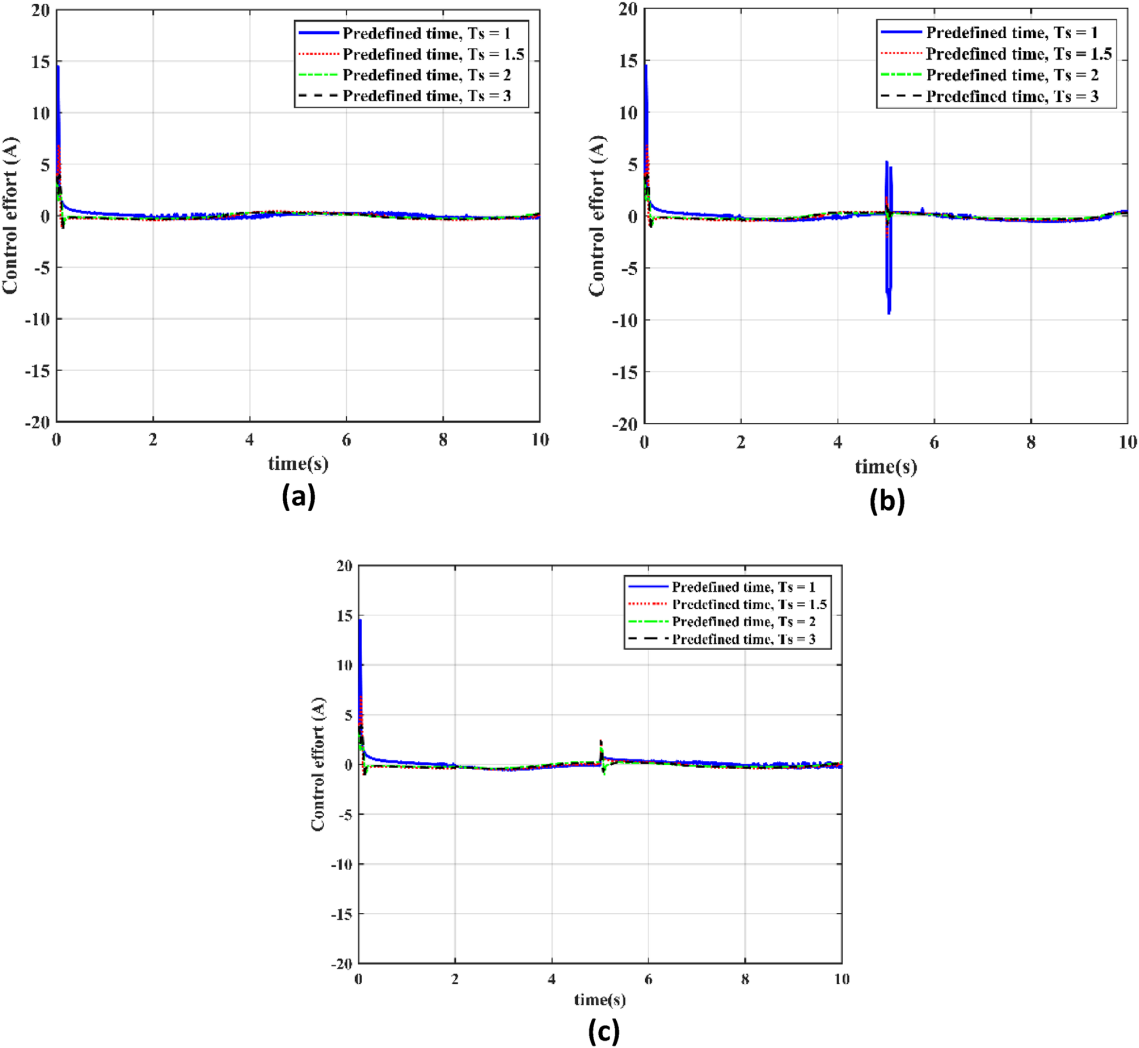
Control effort for the different predefined time settings. (**a**) Control effort for the different predefined time (*Ts*) in Case 1. (**b**) Control effort for the different predefined time in Case 2. (**c**) Control effort for the different predefined time (*Ts*) in Case 3.

Figure 9a–c show how the PTCSMAC control rule performs very well and provides chatter-free control in all predefined time settings under a variety of cyber-attack scenarios. The PTCSMAC's control effort is smooth and consistent in all scenarios attack-free (Case 1), low-level (Case 2), and high-level (Case 3) which suggests that it is robust and successful. The CPS servo system's stability and precision depend on this smooth control, which keeps the actuators safe from chattering's damaging consequences. Together, the results show that our suggested controller not only satisfies the CPS servo system's high-precision control requirements, but also maintains its efficiency and composure over a variety of predetermined times even in the appearance of the most disruptive cyberthreats proves its superiority in control performance.

### CPS servo system's performance analysis under of different activation functions

According to the Table 4, we will use a range of activation functions to conduct an in-depth analysis of the tracking and control performance of the CPS servo system in this section. In particular, we will evaluate how well each activation function performs in maintaining accurate trajectory tracking and seamless control effort in both uncompromised and compromised cyber environments. To determine the advantages and disadvantages of our suggested controller in various functional contexts, the analysis will concentrate on finding any performance imbalances, especially the chattering seen with the Cosine Function in Case 2.

**Table 4:** The choice of different activation function.

Choice of activation function	Mathematical description
Sigmoid function (Sig F)	$G(a,b,x) = \frac{1}{{1 + e\,^{( - a \cdot x + b)} }}$
Hyperbolic tangent function (HTF)	$G(a,b,x) = \frac{{1 - e\,^{( - a \cdot x + b)} }}{{1 + e\,^{( - a \cdot x + b)} }}$
Hard limit function (HLF)	$$G(a,b,x) = \left\{ {\begin{array}{*{20}l} {1,{\text{ if a}}{\text{.x }} \le 0} \hfill \\ {0,{\text{ otherwise }}} \hfill \\ \end{array} } \right.$$
Cosine Fourier basis Function (CF cos )	$G(a,b,x) = {\text{Cos}} \,(a.x + b)$

**Figure 10:**
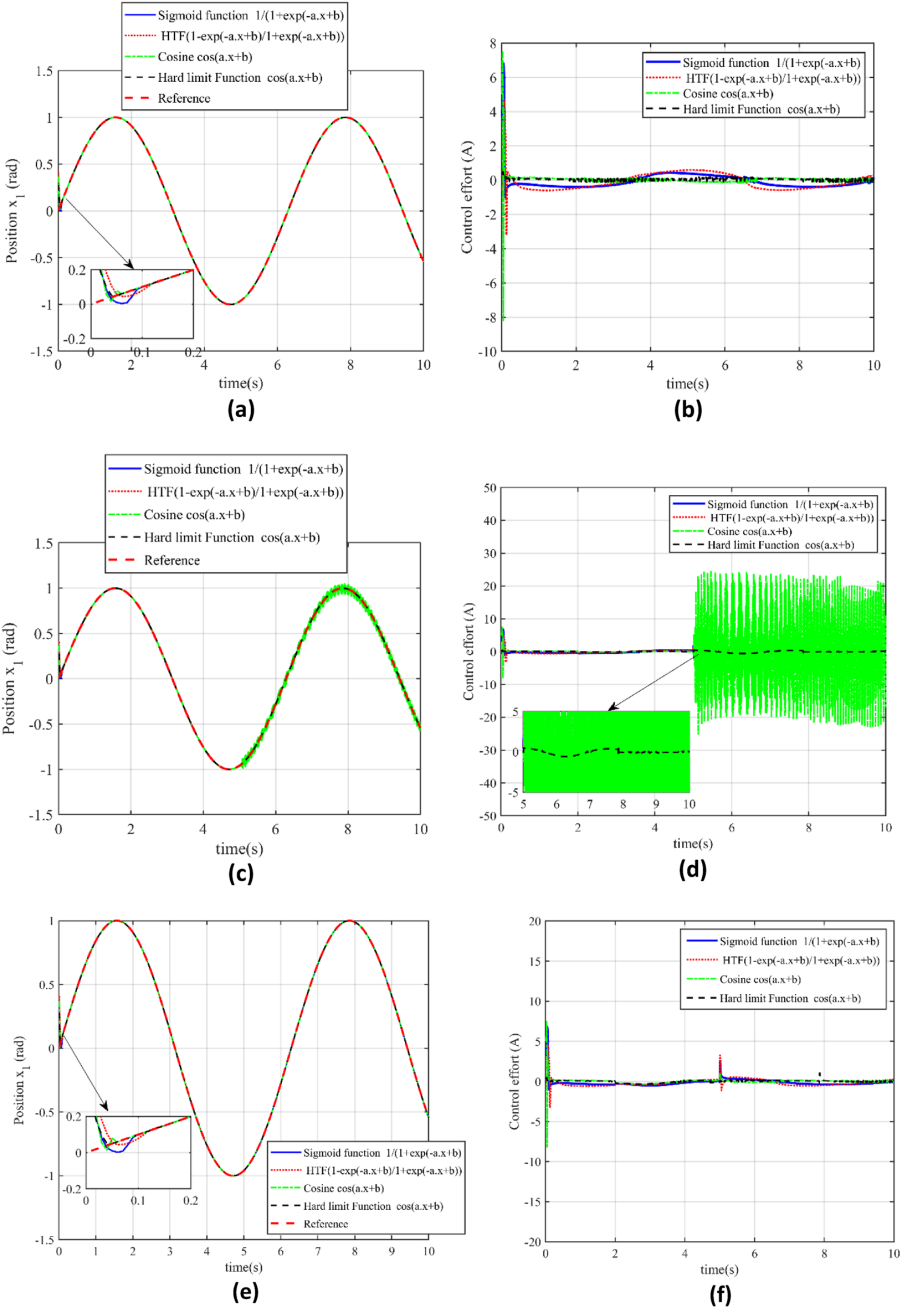
Performance and evaluation of the different activation functions. (**a**) Reference v/s output trajectory tracking for different activation functions in case 1. (**b**) Control effort for the different activation functions in Case 1. (**c**) Reference v/s output trajectory tracking for the different activation functions in case 2. (**d**) Control effort for the different activation functions in Case 2. (**e**) Reference trajectory tracking for the different activation functions in case 3. (**f**) Control effort for the different activation functions in Case 3.

From the above Fig. 10a–f, we can analyze the performance of the CPS servo system under different activation functions, as summarized in Table 4, indicates the system's adaptability and precision across various mathematical models. These functions include the Sigmoid Function (Sig F), Hyperbolic Tangent Function (HTF), Hard Limit Function (HLF), and Cosine Fourier Basis Function (CF cos). The capability of the proposed control to track the desired output trajectory in an attack-free scenario (Case 1) and under cyberattack conditions (Case 2) is evaluated for each activation function. According to our findings presented in Fig. 10, the proposed controller consistently achieves high precision in position tracking for all activation functions, even in severe conditions such as a high-level cyberattack. The control effort remains smooth and free from chattering in all cases, with the exception noted in Fig. 10d for the Cosine Function during Case 2, where some irregularity is observed. Regardless of this exception, our proposed PTCSMAC controller's performance with different activation functions suggests a robust and versatile design, capable of maintaining CPS servo position control system integrity and precision across a range of operational settings.

### Tracking error analysis for the different activation functions in each case

In this section we have demonstrated the error evaluation formulas to assess performance criterion such as Integral square error ,${\text{ISE }} = \int e (t)^{2} \cdot dt$, Integral absolute error , ${\text{IAE }} = \int | e(t)| \cdot dt$ , Integral time absolute error , ${\text{ITAE }} = \int t \cdot |e(t)|dt$, Integral time square error , ${\text{ITSE }} = \int t \cdot \left[ {e(t)^{2} } \right] \cdot dt$ . The tracking error-based performance analysis for the different activation functions in each case is discussed as follows.

**Figure 11:**
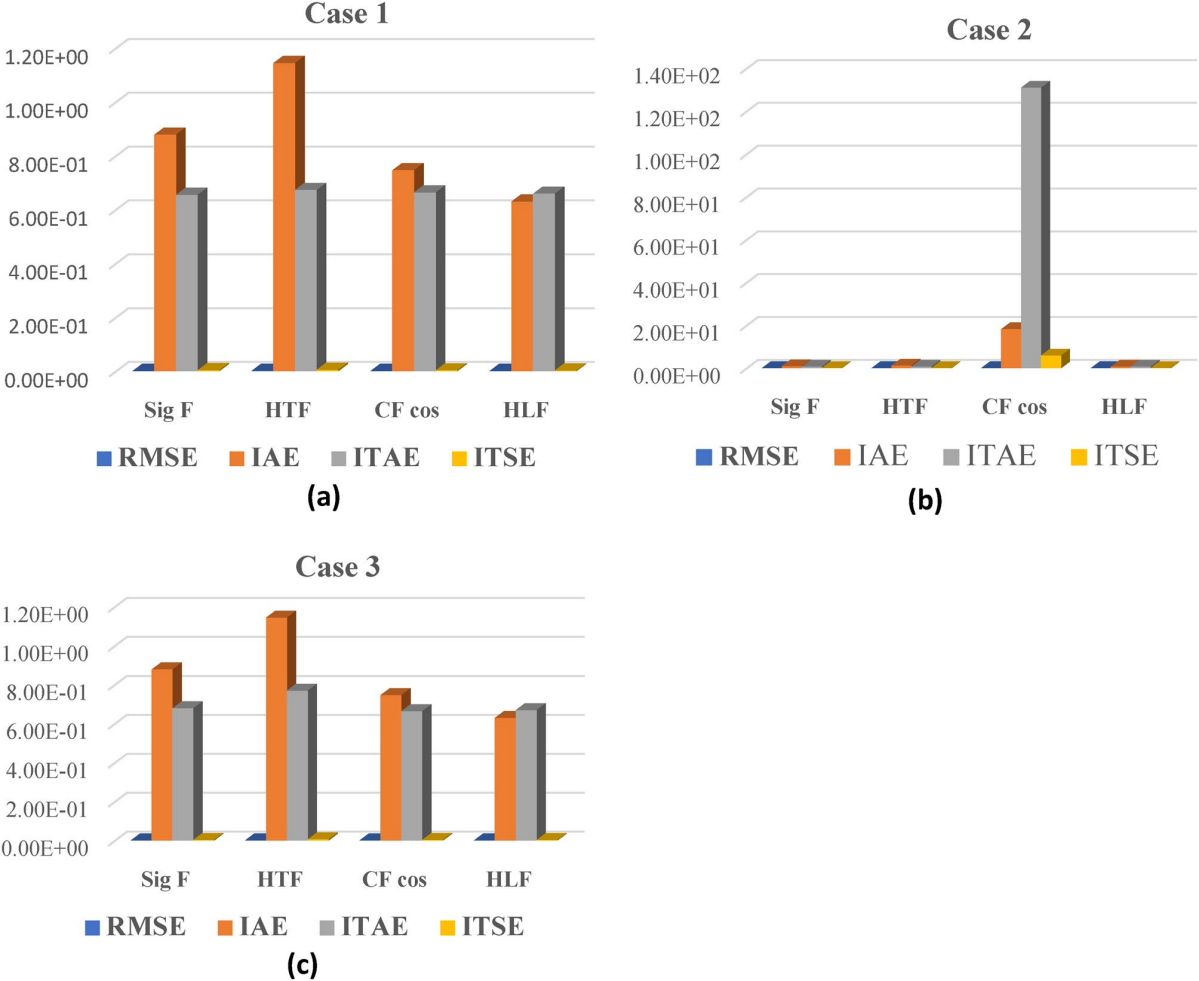
Error performance evaluation for different activation functions under different cyber-attack scenarios. (**a**) Tracking error analysis for the different activation functions in case 1. (**b**) Tracking error analysis for the different activation functions in case 2. (**c**) Tracking error analysis for the different activation functions in case 2.

These are some of the performance indicators RMSE, IAE, ITAE, and ITSE that are utilized to assess the accuracy of the suggested controller with various activation functions for Case 1, an attack-free scenario. The Sigmoid Function (Sig F) showed the lowest root mean square error (RMSE), suggesting excellent position tracking accuracy. In terms of IAE and ITSE, the Hard Limit Function (HLF) performed better than the others as shown in Fig. 11a, indicating that it had the smallest overall error and the least error over time, respectively, demonstrating its effectiveness in rapidly reducing error. Nonetheless, the Sigmoid Function had the lowest ITAE (which accounts for the mistake magnitude over time), demonstrating its efficacy in reducing errors that endure over time.

Case 2: there is a significant increase in the root mean square error (RMSE) of the Cosine Fourier Basis Function (CF cos) under low-level cyberattack conditions was observed in Case 2, suggesting a notable decline in tracking accuracy. The ITAE for CF cos sharply increased as shown in Fig. 11b, indicating the significant impact of error with time, while its IAE skyrocketed, indicating a significant cumulative magnitude of error. When compared to the other functions, the HLF consistently performed well across all metrics, demonstrating its greater resilience to low-level cyberattacks.

In Case 3 (high level attack), showed the RMSE patterns that were comparable to those seen in Case 1, and the Sigmoid Function continued to operate with excellent accuracy. The HLF kept showing the lowest IAE and ITSE, demonstrating its steady performance in reducing errors both instantly and over time as shown in Fig. 11c. The Sigmoid Function had the lowest ITAE, indicating that even in the face of a serious cyberattack, it is still capable of containing errors that build up over time.

These findings show that our suggested controller accomplishes accurate and reliable control in every scenario. Various activation functions have different performance strengths in diverse contexts. The Sigmoid function tends to be better at eliminating time-persistent errors, while the Hard Limit function seems to be more resilient in lowering RMSE and overall errors.

## Conclusion and future directions

The Seeker Coordinator Servo Control systems are often vulnerable to uncertainty and cyber-attacks which are discussed in detail in this study. Simulation verifications demonstrated that the suggested framework, which combines strong control and intrusion ELM estimation techniques, efficiently maintains system performance and security as compared to existing techniques. Future study will concentrate on implementing real-time attack recovery mechanisms and expanding this technique to include more intricate industrial control systems. The main key feature of our proposed control is a simple in structure which is a combination of modified weight updated extreme learning machine (ELM) and an improved PDT convergent sliding mode strategy (SMS). This combination makes the control approach robust and reliable in performance even in the presence of multiple uncertainties. Although seeker coordinator servo CPS nonlinear positioning system is a complex system but our proposed PTCSMAC control law performs best while position tracking even under different cyber-attacks. Thus, our proposed controller has high accuracy and reliability. Our proposed control has been verified through rigorous simulations and comparative analysis. This control method is robust to cope cyberattacks since it is adaptive in nature so it can be used for variety of cyber-physical systems. This can even be used in the presence of high-level cyber-attacks in the CPS servo position control system. The proposed control can also manage a wide range of uncertainties including cyber-attacks which is adaptive to varying system conditions and cyber-attack levels. Our next aims are to design a robust control technique based on Fuzzy logic, ELM based uncertain resilient controller for unmodeled dynamics in CPS systems, adaptive control for CPS malicious attacks and ELM based hybrid control techniques.

## Data Availability

All the data has been included and cited within this article.
